# Expanding applications, accuracy, and interpretation of laser speckle contrast imaging of cerebral blood flow

**DOI:** 10.1038/jcbfm.2015.84

**Published:** 2015-05-06

**Authors:** S M Shams Kazmi, Lisa M Richards, Christian J Schrandt, Mitchell A Davis, Andrew K Dunn

**Affiliations:** 1 Department of Biomedical Engineering, The University of Texas at Austin, Austin, Texas, USA

**Keywords:** camera imaging, laser speckle contrast imaging, laser speckle flowmetry, optical imaging

## Abstract

Laser speckle contrast imaging (LSCI) provides a rapid characterization of cortical flow dynamics for functional monitoring of the microcirculation. The technique stems from interactions of laser light with moving particles. These interactions encode the encountered Doppler phenomena within a random interference pattern imaged in widefield, known as laser speckle. Studies of neurovascular function and coupling with LSCI have benefited from the real-time characterization of functional dynamics in the laboratory setting through quantification of perfusion dynamics. While the technique has largely been relegated to acute small animal imaging, its scalability is being assessed and characterized for both chronic and clinical neurovascular imaging.

## Introduction

Generally, optical techniques for measuring of cerebral blood flow (CBF) either rely on spatio-temporal tracking of blood cells or use of dynamic light scattering (DLS). The former include laser scanning microscopy,^1, 2^ high speed red blood cell (RBC) photography,^3, 4^ and recently RBC-optical coherence tomography.^5^ Dynamic light scattering techniques include laser Doppler flowmetry (LDF),^6, 7^ diffuse correlation spectroscopy,^8, 9^ DLS-optical coherence tomography,^10^ and laser speckle contrast imaging (LSCI). Over the preceding decade, LSCI has become a popular method for visualizing and quantifying neurovascular blood flows in small animals.

This review summarizes the typical instrumentation and image analyses used for LSCI, while surveying existing and emerging applications of speckle imaging studies of CBF. The advantages and limitations of speckle flowmetry are also discussed along with the possibility of quantifying absolute perfusion.

## Laser speckle contrast imaging basics

A camera records a speckle image when a specimen is illuminated with coherent laser light due to the fact that the laser light reaching each pixel has traveled slightly different pathlengths. Time varying changes in that speckle pattern contain information about the motion of the moving particles encountered in the specimen. Laser illuminated images of perfused tissue capture a speckle pattern that when integrated over timescales comparable to the motion dynamics, approximately 1 millisecond for biologic tissues, results in visible blurring. The degree of spatial blurring can be quantified by calculating the speckle contrast, *K*, defined as the ratio of the standard deviation, *σ*_s_, to the mean intensity^11^ of pixel values, <*I*>,


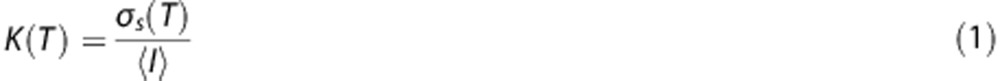


where *T* is the exposure time of the camera. Speckle contrast imagery taken at shorter exposures gives rise to higher contrast and accentuates faster perfusion relative to imagery captured at longer exposure durations.^12, 13^ A pixel-by-pixel speckle contrast calculation can be made temporally from sequentially acquired raw speckle images, or alternatively in a single speckle image, by means of computing speckle contrast in a moving pixel window. The latter is performed by determining the contrast value for the central pixel in an odd numbered *N*x*N* pixel window (typically *N*=7). A statistically comparable temporal contrast calculation would require computation at each independent pixel over *N*^2^ sequential images in time. The spatial analysis of the temporal phenomena assumes equivalence of spatial and temporal (e.g., ergodic) statistics, which breaks down in the absence of flow or with a significant contribution of static scattering but can be accounted for through multiple exposure imaging. For CBF imaging, spatial contrast computation is often used in lieu of temporal contrast to achieve higher imaging speed as only a single image is needed for contrast computation, enabling better temporal resolution for noise reduction and capturing rapid flow dynamics while providing comparable flow indices to temporally computed contrast in the presence of perfusion.^14, 15^ Additionally, LSCI simulations using actual neurovascular anatomies have indicated that a large degree of spatial integration occurs in widefield speckle imaging,^16^ suggesting that imaging regions are sensitive to neighboring and underlying flows that likely obviate the effects of any added blurring (i.e., loss of spatial resolution) from using a spatial window to compute speckle contrast.

**Figure 1:**
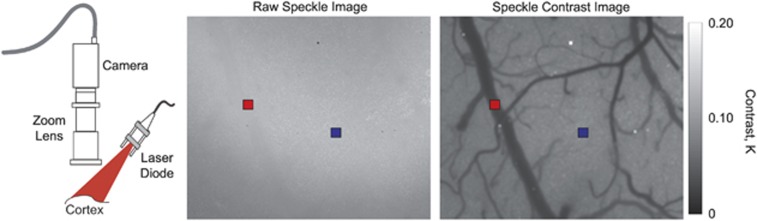
Laser speckle contrast imaging. Remission of scattered laser light from tissue is imaged by a charge coupled device (CCD) camera (left). Image processing with a moving spatial window computes the speckle contrast (*K*) across the raw field-of-view (center) to obtain the speckle contrast image of flow (right). Lower speckle contrast is often observed in pial vessels (red selection) relative to parenchymal regions (blue selection) due to increased blurring of the speckles from faster flows.

A typical example of a raw speckle image of the mouse cortex is shown in Figure 1. The grainy appearance of the speckle pattern is accentuated by some regions appearing more blurred than others. A spatially computed speckle contrast image (using Equation 1) quantifies this blurriness into a qualitative image of perfusion. Large surface vessels are easily resolvable as areas of higher flow indicated by lower speckle contrast values, appearing darker in the speckle contrast image shown (Figure 1). Likewise, LSCI is sensitive to flows in areas of tissue that do not contain resolvable vasculature (i.e., parenchyma) but contain flow information about underlying depth-distributed microvasculature, which typically exhibit higher contrast values due to lower blood flow. Over sequential images, temporal changes in blood flow may be readily observed^17^ across the field of view, especially when flow is interrupted.^18^

## Quantitative imaging of blood flow

Practical speckle imaging is ultimately a time-integrated technique that enables rapid flow visualization by computing an image of speckle contrast (Figure 1) with simple image processing algorithms that can be performed faster than typical camera frame rates.^19^ However, the obtained speckle contrast values, *K*(*T*), are qualitative flow estimates that hinge on the proper selection of the camera exposure duration and the imaging instrumentation. The exact relationship between *K*(*T*) and the underlying blood flow is nonlinear and while it remains an active area of study, several advances and improvements have been made to better estimate flow dynamics that are relevant for monitoring neurovascular flows. Over the years, quantitative models have been used to relate the speckle contrast values to the characteristic correlation time of the speckles, *τ*_c_. The correlation time is inversely related to the rate of light scattering off of the encountered moving particles (e.g., DLS). The inverse of the correlation time is a commonly reported metric by DLS techniques, including LDF and diffuse correlation spectroscopy as well, and principally quantifies the sampled blood flow. Fercher and Briers^20^ proposed a simplified model that related the speckle contrast values to the speckle correlation time, *τ*_c_, which was later expanded upon by Bandyopadhyay *et al*^21^ (Equation 2) by incorporating a more rigorous relationship initially proposed by Goodman.^22^ Therefore, the speckle correlation time can be extracted from the following relation.


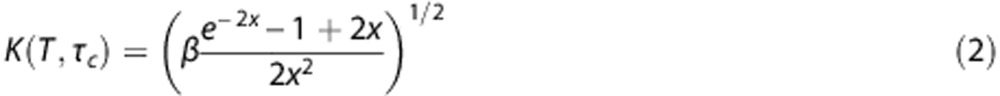


Where *x*=*T*/*τ*_c_. This relationship highlights that the observed contrast is a function of the exposure duration of the camera as well as the speckle correlation time and an instrumentation factor, *β*. This model^21^ and its former variant have been used to predict similar relative CBF dynamics in terms of inverse correlation time (1/*τ*_c_) changes^23^ and collectively have been adopted for a wide range of blood flow imaging applications.

Inverse correlation times (ICTs) from speckle imaging have often been reconciled both theoretically^24^ and empirically^17, 23^ with those measured with LDF. Consequently, the ICT is often interpreted as being proportional to the speed of the moving particles (i.e., blood cells), an assumption retained from LDF measurements of capillary flow.^6^ The exact physical relationship of the ICTs with CBF across both resolvable and unresolvable cortical vasculature requires further examination, but is known to be a function of the specimen properties (i.e., structure and scattering properties) and the imaging geometry (i.e. laser illumination and collection).^6^ In the limiting case of imaging an isolated capillary vessel, the ICT would be a measure of the erythrocyte speed. This assumption may scale to larger vessels if reporting only relative flow dynamics from the same region, which has been shown by a number of calibration studies in single microfluidic flow channels^14, 25, 26, 27, 28^ with comparable sizes to pial microvessels. The changes in the speckle flow indices were found to be significantly proportional to the pump flow dynamics. Therefore, most studies interpret this speckle flow index (i.e., ICT and its equivalent) as the flow velocity of their respective specimens. However, this assumption may become inaccurate in the presence of variations in blood vessel caliber and hematocrit. Additionally, speckle flow indices from parenchymal regions cannot be deterministically decoupled to each contributing microvessel. Nonetheless, strong correlations between speckle-based flow measures and alternative perfusion indices^29, 30, 31, 32^ across single vascular and parenchymal regions suggest that flow dynamics within any given region of interest may be accurately captured. For all practical purposes, the ICT still serves as a regional perfusion index in arbitrary physical units.

## Laser speckle contrast imaging applications in physiologic and preclinical studies

Initial speckle imaging applications to CBF characterization evaluated the ability to measure the hemodynamic response to functional challenges (i.e., electrical stimulation, hypercapnia, and cortical spreading depressions) and physiologic alterations (i.e., vascular occlusions, hyperoxia, and hypothermia) in the rodent and feline cortices.^17, 33, 29, 34, 30, 35, 36^ The focus of many of these pilot studies was an assessment of speckle imaged cortical perfusion dynamics for proof of concept or comparison with a myriad of alternative flowmetry techniques. In particular, Devor *et al*^37^ observed that the rise and increase in speckle predicted functional flows correlated in space and time with arteriolar vasodilation and constriction, confirmed with extensive two-photon microscopy along with multispectral imaging for blood volume and oxygenation metrics. The strong sensitivity to the CBF dynamics from these LSCI studies has subsequently fostered applications in the characterization of neurovascular coupling and disease models. Here, the spatial and temporal resolution of LSCI is often leveraged to obtain functional maps in small animals arising in the form of transient hyperemia with and without resting-state CBF alterations. Prior studies attempting to assess correlation of CBF and neural function have also used laser Doppler techniques,^7, 38^ but the spatio-temporal advantages of widefield LSCI have facilitated regional mapping and monitoring.

The correspondence of the laser speckle observed regional CBF dynamics and those observed with LDF^39^ and evoked potentials were promising indicators for functional mapping and characterization. These studies typically involve immobilization of a subject and are nearly always performed under anesthesia.^33^ The effects of anesthesia selection have recently been examined as well with the aid of LSCI,^40, 41^ particularly by imaging the variations in CBF under both resting-state conditions and during induced flow changes from cortical spreading depressions (CSDs).

Optical probing, perturbation, and microscopy are increasingly being use in tandem for studying neurovascular coupling,^42^ microvascular oxygenation,^43^ and cortical connectivity mapping^44^ in a noncontact manner. Specifically, Scott and Murphy^45^ have examined both direct neuronal stimulation via photostimulation of Channelrhodopsin-2 channels in pyramidal neurons of transgenic mice and peripheral sensory stimulation with both intrinsic optical imaging (i.e., blood volume) and speckle contrast imaging (i.e., blood flow) for elucidating pathways that enable functional hyperemia. Traditionally, monitoring of neuro-electric potentials with electrodes^30, 46^ and blood oxygenation and volume with multispectral or intrinsic imaging^47^ often substantiated the transient hyperemia observed with LSCI. More directly, the use of voltage-sensitive dyes and intrinsic hemoglobin absorption imaging has also been combined with LSCI-based CBF imaging for an all optical characterization of neural-electric phenomena and local hemodynamics.^48^ Recently, the patho-physiological pathways linking CSDs and neuronal stress to the nociceptive signals of migraines are beginning to be elucidated by monitoring flow dynamics in parenchymal and individual cortical microvessels. Specifically, Kartara *et al*^49^ probed these signals with antagonists, histology and *in vivo* imaging of fluorescence markers that were all principally reliant on the LSCI captured CBF dynamics, potentially guiding pharmacological discovery. This is a unique but growing example of noncontact techniques to probe large regions of the intact cortex with the spatial and temporal resolution afforded by widefield speckle imaging in conjunction with other optical microscopy techniques. In several of these implementations, LSCI has been adapted to the existing commercial and custom microscopes as a complementary technique, often sharing the same imaging optics.

## Clinical applications in intraoperative imaging of cerebral blood flow

**Figure 2:**
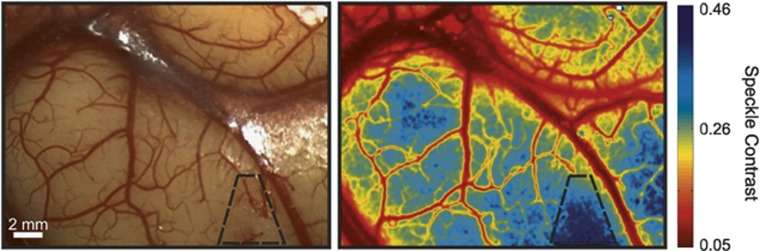
Photograph of human cortex captured by a surgical microscope and the corresponding speckle contrast image. The outlined region has reduced cortical blood flow from vascular cauterization.

Laser speckle contrast imaging has also recently been applied to human cortical blood flow imaging during neuro-surgical procedures. These pilot clinical studies show both the technical adaptation of existing optical microscopes (i.e., surgical microscopes) for laser speckle imaging^50, 51^ and the use of standalone systems.^52, 53^ A primary concern has been assessing cortical vascular patency during the surgical procedures (Figure 2).

Recently, Hecht *et al*^52, 53^ have observed significant increases in LSCI predicted cortical CBF after vascular bypass^52, 53^ as well as from induced hyperemia (CO_2_ challenge).^53^ These sustained and transient hyperemic events were observed in both pial and parenchymal regions of the cortical speckle contrast imagery obtained with a standalone commercial LSCI system (Moor Instruments, Wilmington, DE, USA), where flux was assumed proportional to 1/*K*^2^. Contrastingly, Nomura *et al*^51^ compared intraoperative speckle contrast imagery with pre- and post-operative SPECT (single photon emission computed tomography) in extracranial–intracranial bypass procedures for aneurysm clipping and ischemic cerebrovascular disease. The speckle contrast images were examined before and after macrovascular interruption and anastomosis. While flow reductions from the vascular interruption were comparable between the imaging modalities, the investigators observed weak correlations of parenchymal flow increases from the speckle imagery and SPECT after vascular anastomosis. However, these findings were attributed to the combined effects of intraoperative (e.g., anesthetized) LSCI versus awake SPECT comparisons made 6 months later and the possible disparity between speckle imaging field and regions actually receiving perfusion.

Intraoperatively, indocyanine green fluorescence angiography and videography is often used for verifying vascular patency,^54^ particularly before and after aneurysm trapping or vascular bypass.^55, 56, 57^ A preclinical study in rodents compared LSCI and indocyanine green angiography simultaneously, examining the sensitivity and accuracy of flows in cortical vessels undergoing thrombosis.^31^ Laser speckle contrast imaging was found to better delineate both full and partial occlusion in terms of relative flow magnitudes. Preliminary clinical comparisons have cursorily examined the patency of bypass grafts with positive flow confirmed by both optical techniques (LSCI and indocyanine green angiography),^53^ though imaging obstructions prevented detailed analysis with speckle imaging.

Other novel applications of LSCI during neurosurgery include functional mapping during awake craniotomy,^58^ and visualizing propagation of CSD after malignant stroke.^59^ In the mapping study, preliminary results show a positive correlation between LSCI and electrocortical stimulation, the standard method for intraoperative functional mapping. Specifically, a significant flow increase was measured by LSCI during stimulation of confirmed regions of functional motor areas in six out of eight patients, with no significant change observed in control regions. In the stroke study, LSCI measured the spatio-temporal blood flow responses to the peri-infarct CSDs by characterizing propagation velocity, area, and response phase (i.e., hyperemia, hypoemia, or both) followed by postoperative CSD and infarct monitoring with electrocorticography and serial magnetic resonance imaging, respectively. Beyond technical characterization, these multimodal techniques are aimed at linking the intraoperative intervention to chronic monitoring of the pathology and potential recovery.

In applications such as clinical intraoperative imaging, both cardiac and respiratory motion artifacts have a large impact on image sets acquired over several minutes. Cardiac motion artifacts can be corrected using an *ad hoc* filter generated using the ECG waveform recorded during image acquisition.^50^ Respiratory and tissue motion artifacts can be corrected using image registration, which eliminates image displacement over time and facilitates regional tracking of CBF temporally.^60^ The CBF noise over time was measured to be as low as 6% on average when ECG filtering and image registration is performed together, and as high as 25% on average without these corrections.^60^

## Technical characterization, improvements, and emerging applications

### Multiexposure Speckle Imaging

**Figure 3:**
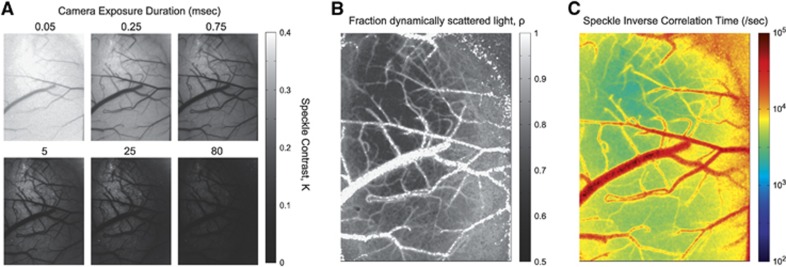
(**A**) Speckle contrast images at six separate exposures exhibiting increased speckle visibility (e.g., contrast) at lower exposures and highlighting varying flow range sensitivity with exposure selection. (**B**) Fraction of collected light at each pixel scattered by moving particles (i.e., dynamically scattered light, *ρ* of Equation 3) and (**C**) corresponding inverse correlation time map extracted from fitting speckle contrast relationship with exposure duration, *K*(*T*), to the visibility expression in Equation 3.

With the need to image large microcirculatory flow ranges in small animals and humans, expanded calibration studies particularly suited for neurovascular flows are increasingly being performed.^14, 18, 25, 26^ Most of these studies have highlighted LSCI sensitivity to the camera exposure duration. In particular, shorter exposures are sensitive to faster flows and thereby reciprocally, longer exposures to slower flows (Figure 3A). Therefore, the selection of the camera exposure duration directly influences the accuracy of the flow predictions. To avoid repeated *ex vivo* calibrations, multiexposure speckle imaging (MESI) has been proposed to map the dependence of the speckle contrast value on the exposure duration of the camera.^14, 61, 62^

By accounting for exposure dependence, the instrument factors, such as *β* (Equations 2 and 3), can then be estimated regardless of speckle sampling, enabling a more absolute estimate of ICT, the principal DLS flow index. One significant advancement is the ability to decouple the effects of light that has been scattered by both moving and relatively stationary particles, known as heterodyne mixing.^6^ Specifically, the MESI speckle visibility model is expressed as,


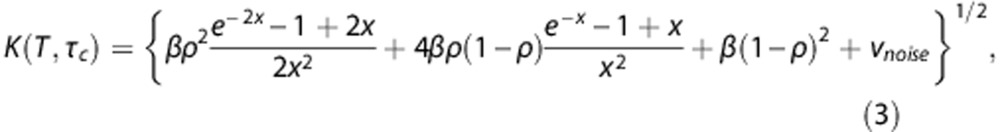


where again 
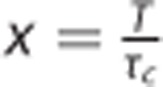
 and *β* is again a normalization factor to account for speckle sampling, *ν*_noise_ models instrument noise and *ρ* is the fraction of light dynamically scattered.^14^ The value of *ρ* is indicative of the fraction of the sampled tissue that was actively perfused regardless of flow magnitude, akin to a volume fraction of flowing blood, from the perspective of the camera. This parameter is particularly necessary for examining parenchymal flows and those captured through thinned skull preparations,^50, 63^ while less impactful for quantifying flows from exposed (e.g., pial) vessels where nearly all the captured light has been scattered by moving particles (*ρ*→1, Figure 3B) and Equation 3 approximates to Equation 2. Practically, the value of *ρ* can be used automate the segmentation of the cortical speckle contrast imagery into parenchymal and pial vascular compartments (Figure 3B) that may be useful for subsequent analyses. Modifications to the speckle imaging DLS models afforded by MESI (e.g., Equation 3) enable better uncoupling of the flow related contributions to the imaged speckles. This is principally portrayed in terms of an ICT map that is quantitatively illustrative of the perfusion magnitude (Figure 3C). *In vitro* and *in vivo* validations^18, 27, 32, 50, 64^ of MESI show convincing evidence of substantial improvements in the quantitative accuracy and fidelity of the speckle flow measurements, though the detailed evaluations have been mainly been limited to resolvable cortical vasculature (i.e., pial vessels). Nevertheless, the technical validations show strong calibrations over a wide range of flows that can be tuned by inclusion of shorter or longer exposures,^13^ typically spanning from the 10^−5^ to 10^−1^ seconds for small animals, and suggest improved accuracy in registering the true rate of the imaged motion (e.g., CBF dynamics).

## Chronic monitoring of blood flow

The monitoring of sustained flow alterations has pushed LSCI application further into longitudinal studies. Particularly, stroke studies rely on the intrinsic contrast obtained from imaging the effects of DLS without introducing exogenous contrast agents into the bloodstream^65^ over timescales ranging from a few days to several weeks. However, speckle imaging largely has been limited to acute studies of neurovascular disease models.^17, 29, 30, 34, 66^ The utility of LSCI in stroke studies has ranged from more qualitative visualization^44, 66^ of the cortical microvessels and their collateral flow redistribution, to using DLS models to provide quantitative monitoring of flow deficits, ischemia, and associated transients.^46^ Typically, single exposure speckle imaging has been extended for intermittent monitoring of cortical flow reductions, remodeling,^66^ and the effects of animal preconditioning^67^ up to a period of 24 hours.

**Figure 4:**
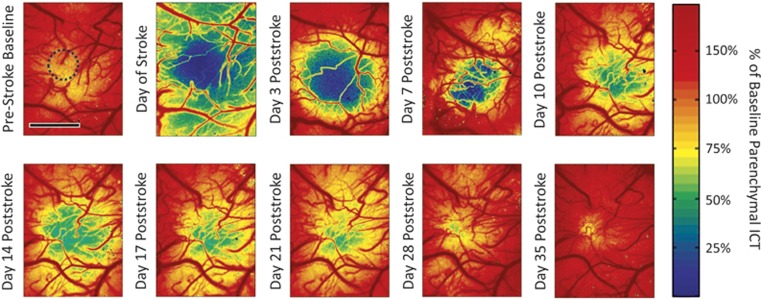
Cortical blood flow dynamics chronically captured before and after regional photo-thrombosis (outlined region) with periodic multiexposure speckle imaging in a cranial window implanted mouse. Photothrombotic parameters: 20 mW of 532 nm wavelength laser, spot diameter of 600 *μ*m, 15 minutes of irradiation with Rose Bengal administration (100 mg/kg, intraperitoneal). Image scale bar=500 *μ*m. ICT, inverse correlation time.

Multiexposure speckle imaging has shown to be well suited for imaging a wide range of baseline and induced flow changes over several imaging sessions.^32^ It confers the ability to quantify a large range of resting state flows as well as reductions and elevations, and therefore benefits studies reliant on capturing infarct size and severity. By having a quantitative CBF baseline, chronic flow dynamics can be obtained to examine the effects of lesion models that may elicit sweeping changes in blood flow beyond the sensitivity of a single camera exposure duration.^32^ Specifically, MESI flow imagery can quantify large regional perfusion deficits and subsequent increments (Figure 4) over a period of several weeks and potentially months in single animals. Such monitoring has implications for studying functional plasticity and tissue health through the flowing phase of vascular remodeling and reperfusion. Inverse correlation time measurements in Figure 4 were taken before and after a photo-thrombotic occlusion targeting both pial vessels and parenchyma nonspecifically. The occlusion paradigm leads to a large scale parenchymal flow reduction acutely that is quickly resupplied followed by a sustained flow deficit that chronically recovers. The technical advances in speckle imaging have enabled improved quantification of the encountered DLS. However, interpreting or relating these metrics (relative ICT images of Figure 4) to the underlying perfusion requires characterization of both the spatial integration and the degree of flow sensitivity observed with LSCI, particularly with respect to the parenchymal or subsurface microcirculation. Only then, can more detailed pathologic classification be facilitated, such as monitoring the boundaries of the ischemic core and penumbra in Figure 4.

## Interpreting speckle images of neurovascular flows

**Figure 5:**
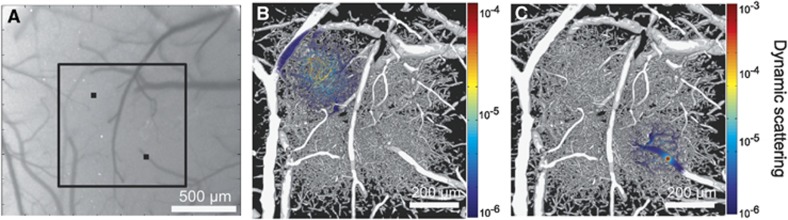
(**A**) Speckle contrast imaging of mouse cortex. (**B** and **C**) Two photon imaging obtained projection of fluorescently labeled microvasculature (gray scale) used for Monte Carlo modeling of light collection by a camera pixel. Spatial distribution of dynamic scattering (color overlay) encountered by light collected at pixels over parenchymal (**B**) and pial (**C**) vasculature identified in (**A**).

The depth dependence and spatial integration volume of speckle contrast imaging have been only recently begun to be addressed in detail.^16^ Three-dimensional Monte Carlo modeling of the light propagation shows that pixels over a resolvable surface or pial vessels record light that has largely been dynamically scattered within those vessels (Figures 5A and 5C). Pixels over parenchymal regions (Figures 5A and 5B) sample a much wider and deeper interaction volume of depth-distributed microvasculature. The penetrating and ascending arterioles and venules also confine the scattering contributions to those individual vessels due to higher scattering in both magnitude (i.e., multiple scattering) and directedness of blood relative to the neuropil. These interaction volumes suggest potential flow calibration of the surface vascular flows, but greater uncertainty in absolute flow quantification due to the heterogeneity of light scattering interactions in the parenchymal tissue. The regional sensitivity of flow dynamics assessed from parenchymal regions will also be influenced by local vascular density and calibers, ultimately biased by larger magnitude flows when in close proximity. Functional hyperemia is often examined exclusively in the parenchymal space and should evaluate regions distal from pial vasculature to establish better specificity to the underlying cortical flow recruitment. A parenchymal pixel samples a specimen volume that is relatively large both laterally and in depth, encompassing many spatially distributed microvessels (Figures 5A and 5B). A substantial change in flow in a single parenchymal vessel may not manifest as a significant change in the speckle imagery. Therefore, parenchymal flow sensitivity will be limited until the flow change occurs over a substantial portion of the sampled vasculature. Variations in hematocrit can also be large within the microcirculation. However, the corresponding variations in scattering properties that directly impact the speckle flow sensitivity in the pial microvessels are substantially less varied in magnitude.^68, 69^ These interaction volume and specimen factors will likely affect the flow analyses between different regions of interest in the field of view, and can be mitigated by keeping LSCI flow analysis across resolvable microvasculature separate from parenchymal regions.

Generally, these factors suggest that multiple scattering by moving particles is being integrated in the paths of the backscattered laser light that form the speckle image. While speckle sensitivity has been examined in detail with erythrocyte speeds from 0 to 15 mm/s,^13^ the light scattering interrogations are highlighting that in addition to particle speed there is a sensitivity to the number of moving scatterers (e.g., erythrocytes). This is resulting in reinterpretations of the ICTs from being proportional solely to erythrocyte speeds, an assumption retained from LDF, to incorporating integrations factors proportional to the encountered erythrocyte density.^32, 70^

**Figure 6:**
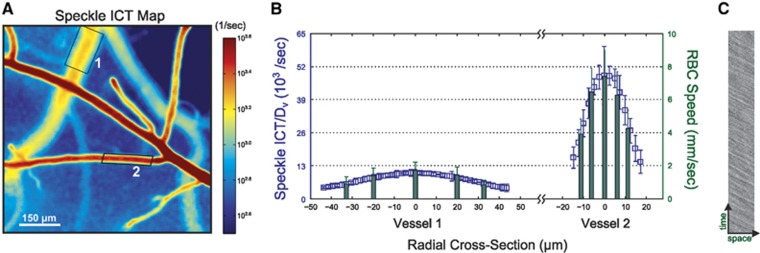
(**A**) Multiexposure speckle imaging (MESI) inverse correlation time (ICT) images of the mouse cortex highlighting radial vascular profiles irrespective of caliber variations between vessels. (**B**) Vessel diameter, *D*_v_, corrected ICTs register consistent comparison of red blood cell (RBC) speeds across pial microvasculature in the mouse cortex when compared with RBC speeds obtained from high speed reflectance imaging (**C**).

Across pial vessels, the relative spatial integration may be estimated by the vessel caliber variations, which can be used to correct the ICTs (Figure 6A) for improved erythrocyte speed comparisons across the resolvable vasculature, exemplified by the speed profiles in Figure 6B. If speckle ICTs are left uncorrected, then a vessel with high erythrocyte density and low velocity may result in comparable speckle ICTs with a vessel of small erythrocyte density and high velocity. Physiologically, the relative volumetric flux though such vessels may by extension be accurately estimated by the product of ICT and the vessel caliber. These observations and correction factors highlight that the governing speckle visibility expressions were principally derived with the assumption that light returning to a camera pixel had interacted with a single moving particle.^11, 21, 50^ A reexamination of the accuracy and the relevance of such derivational assumptions is therefore necessary.

## Instrument configuration and suggestions

The typical LSCI instrument consists of a high coherence laser beam with stable optical power, which is expanded to illuminate an area of interest ranging from a few millimeters to several centimeters. The incidence angle on the sample ranges from near normal to more oblique to reduce specular reflections. The wavelength, *λ*, is generally in the red to near infrared (*λ* ~600 to 800 nm) for tissue scattering to significantly outweigh absorption effects. Typically, diode lasers are the common illumination sources for LSCI, as they provide sufficient optical power ranging from 10 to 10^2^ mW for illuminating cortical fields of view up to a couple of centimeters (i.e., ~5 mW/cm^2^ at 5 msec camera exposure). The coherence length of the laser governs the largest light scattering paths in the tissue that can sample blood flow, and is sufficiently long enough for small animal CBF imaging given that lasers with narrow spectral bandwidths relative to their central wavelength, denoted generally as single longitudinal mode or narrow linewidth lasers, are utilized. For multiexposure systems, a laser amplitude modulator is often added to the optical system to adjust the laser intensity for illuminating the cortex at different camera exposures.^14, 32^ This amplitude modulation can be accomplished through manual adjustment of optical filters or quickly automated with electronically controlled optical modulators. Alternatively, changing the drive current of the laser directly may have detrimental effects on the comparability of the speckle images due to the changes in the laser temporal mode, though exceptions have been noted with some multiexposure implementations.^64^

The second component of the imaging system is the collection optics, usually consisting of objective and tube lenses. Optics are selected to accommodate the desired magnification, *M*, and light collection of the imaging system. The latter is principally determined by the numerical aperture, NA, or f-number (f/# ≈ 1/(2NA)) of the objective lens or lens system. The NA also determines the diffraction limited speckle size,^71^ the radius of which is *r*_s_≈0.6(1+M)λ/NA. The speckle size scales the minimum window size for spatial contrast calculation, and thereby determines the maximum observable contrast value and effective resolution of the LSCI system. Some LSCI implementations attempt to match the imaged speckle size to the pixel size of the camera. This serves as an instrumentation control that enables higher spatial resolution and more accurate estimation of the absolute perfusion with less calibration.^72^ Alternative perspectives suggest two pixel imaging of speckles as being more optimal^73^ for accurately sampling the speckles and is sufficient for estimating ICT variations for monitoring relative CBF dynamics. However, such implementations would require calibration or multiexposure implementations for obtaining absolute ICTs.

The third main component of the LSCI system is the camera on which the scattered and collected light from the sample is imaged to enable recording of the speckle pattern. The specifications of the monochrome cameras used for LSCI vary widely, but uncooled^19, 74^ and inexpensive cameras^75^ in monochrome mode have been shown to provide excellent blood flow imagery and are only differentiated by the time and spatial averaging needed to achieve the desired signal-to-noise. These factors ultimately govern the temporal and spatial resolution practically achievable for monitoring CBFs and are determined by the quantum efficiency of the camera sensors in registering the imaging light as well as the camera frame rate. Maximizing the camera frame rate enables greater temporal averaging of noise depending on the minimum tolerable temporal resolution for the flow measurements. Complementary metal-oxide semiconductor image sensors typically have slightly better frame rate performance, but charge coupled devices are commonly used as well. Selecting cameras with pixel fill factors (i.e., light sensitive region of pixel versus entire pixel area) close to 100% helps improve the signal-to-noise ratio of the light detection and ensures that the imaged speckles get adequately captured by the imaging sensor. New commercial cameras are featuring near-infrared enhanced sensors that may be 3 to 5 times brighter than monochrome cameras used in the past. Ultimately, these signal-to-noise ratio concerns may be compensated for by optimizing the illumination and light gathering power of the optical imaging system as well.

The last component is the control hardware consisting of a computer for processing and displaying the speckle imagery and data acquisition devices for triggering and monitoring signals from the camera and other electronic equipment. Detailed classification analysis remains for LSCI determined flow dynamics to be used as acute and prognostic markers of tissue health. A survey of the custom and commercial LSCI systems utilized by studies referenced in this review suggests that minor variations in wavelength and camera exposure duration exist. However, distinct variations remain in how the raw imagery is subsequently processed, namely using speckle contrast alone (Equation 1) or estimating ICTs using DLS models (Equations 2 and 3). The latter has shown substantially improved flow characterization. As a whole, the surveyed LSCI studies suggest that regional CBF measurements can be made with relatively simple instrumentation. However, the efficacy of quantitative analysis has taken two extremes. One focuses on simplified models to facilitate ease of analysis and others focus on robust accuracy of measurements in quantifying the DLS. Both, however, are subject to relating these measures to the physical phenomena of the blood flow.

Optical access is also necessary for obtaining CBF imagery. Typically, craniotomy and craniectomy procedures establish a cranial window for performing optical imaging. Thinned skull preparations introduce more static scatterer artifacts in the obtained speckle imagery, which reduces the dynamic range of flow related speckle blurring that can be imaged. These static scattering artifacts can be mitigated by performing temporal contrast,^15^ spatio-temporal contrast^76^ analysis, and MESI. However, the use of the former two procedures alone cannot address static scattering that may be interlaced with dynamic scattering within the depths of the tissue. Multiple exposure implementations (Equation 3) are then needed to effectively take into account these added variations for better extracting the motion.

## Advantages

The principal advantage of LSCI is the relative simplicity and cost-effectiveness of its instrumentation for standalone systems or adaptation to common widefield microscopes, particularly when compared with scanning laser Doppler, optical coherence tomography, intrinsic or fluorescent-based RBC tracking, positron emission tomography, and autoradiographic methods. Widefield LSCI of cortical flows in awake and ambulatory rodents has recently been performed,^28^ demonstrating the technical ability to meet small system form factors and accommodation of study designs looking for behavioral correlates. Laser speckle contrast imaging does not require sophisticated equipment and contrast agents, aided by advancements in machine vision, computing power and reliance on optical scattering-based contrast.

There are no specific spatial resolution limitations for maintaining accuracy of the speckle contrast perfusion measurements. Therefore, fields of view can easily scale and spatial resolution can vary from the size of the pixel in object plane (i.e., on the specimen), or in the case of spatial contrast, the *N*x*N* size of the contrast bin in the object plane. Temporal resolution is also well suited to capture CBF dynamics with proper camera and analysis selection, even after typical frame averaging (~10 Hz effective frame rate). While the images are of a two-dimensional plane of focus, the effective three-dimensional interaction volume of the laser light exiting the tissue is often much greater. For functional imaging of the cortical microcirculation, this means that perfusion information comes from areas much larger than the surface image would depict, particularly for the parenchyma. This is an advantage in that detailed depth-resolved imagery and its substantially larger data volume and processing throughput are not necessary to effectively probe functionally sized regions of the cortex in ensemble.

The interactions of the laser illumination in the red and near infrared wavelengths (600 to 800 nm) with the specimen are ultimately reflective and not absorptive, designed to maximize the amount of backscattered or remitted laser light for imaging. Over typical laser powers (10 to 100 mW) and areas of illumination (10^−2^ to 10^1^ cm^2^), this means there is little energy deposition into the tissue avoiding any disruptive effects on the physiology, typically of concern in flow imaging with absorption or fluorescence contrast.

Additionally, the persistent need for relatively noninvasive assessment of CBF over functionally sized regions of the cortex performed repeatedly in the same animal or even across animals may be possible with improved quantitative LSCI techniques, such as multiexposure implementations. Advances in computing power, opto-electronics (i.e., laser powers, form factors, and modulation), and machine vision are making these implementations more feasible.

## Disadvantages

The technical advantages of LSCI are ultimately juxtaposed with the ambiguity in its spatial specificity leading to uncertainties in how to translate its principal flow index, ICTs, into absolute measures of CBF. While spatial integration may be beneficial, the degree that parenchymal imaging can specify subcortical perfusion levels and dynamics may require sample specific calibrations. Some emerging studies are utilizing histologic correlations to assess subsurface sensitivity to large flow changes.^67^ However, ICTs, and to some extent speckle contrast values, are still commonly interpreted as RBC speed,^62^ but the physical reality and manner of multiple dynamic scattering will likely affect the interpretation. However, without detailed characterization of the specimen-specific three-dimensional vascular architecture,^16^ the exact physical relationship of ICTs to both blood volume and absolute flow may remain confounding.

More elaborate implementations and calibrations to aid the accurate extraction of blood flow dynamics from imaged speckles require greater computational complexity (i.e., multiexposure imaging). The feasibility of these systems will be dependent on the application and technical optimizations,^13^ which will likely drive study designs. The combination of alternative optical techniques using coherence gating, such as Doppler-^10^, RBC-^5^, with DLS-^77^ optical coherence tomography may provide accurate and depth-resolved extraction of flow magnitude and direction from the cortical space. However, the complexity and degree of adaptability of these techniques to existing optical set-ups may be considerable.

Physiologic motion artifacts may of concern in some studies, including anesthetized^78^ but more so awake, freely moving animals.^74^ In anesthetized preparations, cardiac and respiratory motion can affect image quality adversely, exhibited by greater image noise and reduced spatial resolution. However, tissue motion in anesthetized small animals is often tolerably controlled by craniectomy preparations that restore intracranial pressure and stereotaxic immobilization. In such cases, motion correction is often not needed. In freely moving animals, image registration eliminates image displacement over time, producing stable speckle contrast values that can facilitate comparisons over several minutes.^74^ These corrections can be time consuming, especially with handling deformations, and may be prohibitive for real-time flow monitoring. Motion that displaces tissue faster than the camera exposure and comparably as far as blood flow associated displacements can become significantly convoluted with the blood flows. These motion sources cannot be separated from one another, which may limit the range of utilizable exposures and consequently flow sensitivity.

## Conclusions

The advantages of LSCI in general can address unmet technical needs ranging from label-free microvascular angiography to quantifying cortical blood flows, vascular patency, and functional mapping. Laser speckle contrast imaging is based on estimating the temporal dynamics of the scattered laser light, the rate of which is the ICT. Obtaining an accurate estimate of the ICT from speckle contrast imaging is the principle determinant of the accuracy of the CBF quantification, which has been substantially aided with development of MESI. As the flow and spatial sensitivity limits of LSCI are further evaluated and defined, methodological expansion of study designs for cross-specimen and longitudinal imaging of absolute and relative CBF may be possible. By extension, the relation of ICT with volumetric flow and erythrocyte velocity is being examined with substantial calibration ability to units of CBF (volume/time) in pial microvasculature, and more nuanced cross-comparability within parenchymal regions. Ultimately, the desired sensitivity to flow changes, speed of quantification, and physiologic interpretability of the imagery will govern the technical implementation employed and its subsequent analysis.

## References

[bib1] 1Roe AW. Imaging the brain with optical methods. Springer: Washington DC, 2009.

[bib2] 2Kleinfeld D, Mitra PP, Helmchen F, Denk W. Fluctuations and stimulus-induced changes in blood flow observed in individual capillaries in layers 2 through 4 of rat neocortex. Proc Natl Acad Sci USA 1998; 95: 15741–15746. 9861040 10.1073/pnas.95.26.15741 PMC28114

[bib3] 3Duncan DD, Lemaillet P, Ibrahim M, Nguyen QD, Hiller M, Ramella-Roman J et al. Absolute blood velocity measured with a modified fundus camera. J Biomed Opt 2010; 15: 056014. 21054108 10.1117/1.3494565 PMC2966492

[bib4] 4Ishikawa M, Sekizuka E, Shimizu K, Yamaguchi N, Kawase T. Measurement of RBC velocities in the rat pial arteries with an image-intensified high-speed video camera system. Microvasc Res 1998; 56: 166–172. 9828154 10.1006/mvre.1998.2100

[bib5] 5Lee J, Wu W, Lesage F, Boas DA. Multiple-capillary measurement of RBC speed, flux, and density with optical coherence tomography. J Cereb Blood Flow Metab 2013; 33: 1707–1710. 24022621 10.1038/jcbfm.2013.158 PMC3824190

[bib6] 6Bonner R, Nossal R. Model for laser Doppler measurements of blood flow in tissue. Appl Opt 1981; 20: 2097–2107. 20332893 10.1364/AO.20.002097

[bib7] 7Steinmeier R, Bondar I, Bauhuf C, Fahlbusch R. Laser Doppler flowmetry mapping of cerebrocortical microflow: characteristics and limitations. NeuroImage 2002; 15: 107–119. 11771979 10.1006/nimg.2001.0943

[bib8] 8Boas DA, Yodh AG. Spatially varying dynamical properties of turbid media probed with diffusing temporal light correlation. J Opt Soc Am A 1997; 14: 192–215.

[bib9] 9Durduran T, Yodh AG. Diffuse correlation spectroscopy for non-invasive, micro-vascular cerebral blood flow measurement. NeuroImage 2014; 85: 51–63. 23770408 10.1016/j.neuroimage.2013.06.017 PMC3991554

[bib10] 10Srinivasan VJ, Atochin DN, Radhakrishnan H, Jiang JY, Ruvinskaya S, Wu W et al. Optical coherence tomography for the quantitative study of cerebrovascular physiology. J Cereb Blood Flow Metab 2011; 31: 1339–1345. 21364599 10.1038/jcbfm.2011.19 PMC3130321

[bib11] 11Briers JD, Webster S. Laser speckle contrast analysis (LASCA): a nonscanning, full-field technique for monitoring capillary blood flow. J Biomed Opt 1996; 1: 174–179. 23014683 10.1117/12.231359

[bib12] 12Yuan S, Devor A, Boas DA, Dunn AK. Determination of optimal exposure time for imaging of blood flow changes with laser speckle contrast imaging. Appl Opt 2005; 44: 1823–1830. 15813518 10.1364/ao.44.001823

[bib13] 13Kazmi SMS, Balial S, Dunn AK. Optimization of camera exposure durations for multi-exposure speckle imaging of the microcirculation. Biomed Opt Express 2014; 5: 2157. 25071956 10.1364/BOE.5.002157 PMC4102356

[bib14] 14Parthasarathy AB, Tom WJ, Gopal A, Zhang X, Dunn AK. Robust flow measurement with multi-exposure speckle imaging. Opt Express 2008; 16: 1975–1989. 18542277 10.1364/oe.16.001975

[bib15] 15Cheng H, Yan Y, Duong TQ. Temporal statistical analysis of laser speckleimages and its application to retinal blood-flowimaging. Opt Express 2008; 16: 10214–10219. 18607429 10.1364/oe.16.010214 PMC2900841

[bib16] 16Davis MA, Kazmi SMS, Dunn AK. Imaging depth and multiple scattering in laser speckle contrast imaging. J Biomed Opt 2014; 19: 086001. 25089945 10.1117/1.JBO.19.8.086001 PMC4119427

[bib17] 17Dunn AK, Bolay H, Moskowitz MA, Boas DA. Dynamic imaging of cerebral blood flow using laser speckle. J Cereb Blood Flow Metab 2001; 21: 195–201. 11295873 10.1097/00004647-200103000-00002

[bib18] 18Parthasarathy AB, Kazmi SMS, Dunn AK. Quantitative imaging of ischemic stroke through thinned skull in mice with MultiExposure Speckle Imaging. Biomed Opt Express 2010; 1: 246–259. 21258462 10.1364/BOE.1.000246 PMC3005179

[bib19] 19Tom WJ, Ponticorvo A, Dunn AK. Efficient processing of laser speckle contrast images. IEEE Trans Med Imaging 2008; 27: 1728–1738. 19033089 10.1109/TMI.2008.925081

[bib20] 20Fercher AF, Briers JD. Flow visualization by means of single-exposure speckle photography. Opt Commun 1981; 37: 326–330.

[bib21] 21Bandyopadhyay R, Gittings AS, Suh SS, Dixon PK, Durian DJ. Speckle-visibility spectroscopy: A tool to study time-varying dynamics. Rev Sci Instrum 2005; 76: 093110.

[bib22] 22Goodman JW Statistical optics. (1985). Available at <http://adsabs.harvard.edu/abs/1985wi..bookQ..G>.

[bib23] 23Wang Z, Hughes S, Dayasundara S, Menon RS. Theoretical and experimental optimization of laser speckle contrast imaging for high specificity to brain microcirculation. J Cereb Blood Flow Metab 2006; 27: 258–269. 16804551 10.1038/sj.jcbfm.9600357

[bib24] 24Briers JD. Laser Doppler and time-varying speckle: a reconciliation. J Opt Soc Am A 1996; 13: 345–350.

[bib25] 25Choi B, Ramirez-San-Juan JC, Lotfi J, Stuart Nelson J. Linear response range characterization and *in vivo* application of laser speckle imaging of blood flow dynamics. J Biomed Opt 2006; 11: 041129–041129–7. 16965157 10.1117/1.2341196

[bib26] 26Choi B, Ringold TL, Kim J. Methods to enhance laser speckle imaging of high-flow and low-flow vasculature. Conf Proc IEEE Eng Med Biol Soc 2009; 2009: 4073–4076. 19964103 10.1109/IEMBS.2009.5333204 PMC3488601

[bib27] 27Nadort A, Woolthuis RG, van Leeuwen TG, Faber DJ. Quantitative laser speckle flowmetry of the *in vivo* microcirculation using sidestream dark field microscopy. Biomed Opt Express 2013; 4: 2347. 24298399 10.1364/BOE.4.002347 PMC3829532

[bib28] 28Li H, Liu Q, Lu H, Li Y, Zhang HF, Tong S et al. Directly measuring absolute flow speed by frequency-domain laser speckle imaging. Opt Express 2014; 22: 21079–21087. 25321308 10.1364/OE.22.021079

[bib29] 29Ayata C, Dunn AK, Gursoy-OZdemir Y, Huang Z, Boas DA, Moskowitz MA et al. Laser speckle flowmetry for the study of cerebrovascular physiology in normal and ischemic mouse cortex. J Cereb Blood Flow Metab 2004; 24: 744–755. 15241182 10.1097/01.WCB.0000122745.72175.D5

[bib30] 30Strong AJ, Bezzina EL, Anderson PJ, Boutelle MG, Hopwood SE, Dunn AK et al. Evaluation of laser speckle flowmetry for imaging cortical perfusion in experimental stroke studies: quantitation of perfusion and detection of peri-infarct depolarisations. J Cereb Blood Flow Metab 2005; 26: 645–653. 10.1038/sj.jcbfm.9600240 16251884

[bib31] 31Towle EL, Richards LM, Kazmi SMS, Fox DJ, Dunn AK. Comparison of indocyanine green angiography and laser speckle contrast imaging for the assessment of vasculature perfusion. Neurosurgery 2012; 71: 1023–1030, discussion 1030–1031. 22843129 10.1227/NEU.0b013e31826adf88 PMC3718844

[bib32] 32Kazmi SMS, Parthasarthy AB, Song NE, Jones TA, Dunn AK. Chronic imaging of cortical blood flow using Multi-Exposure Speckle Imaging. J Cereb Blood Flow Metab 2013; 33: 798–808. 23571277 10.1038/jcbfm.2013.57 PMC3677120

[bib33] 33Durduran T, Burnett MG, Yu G, Zhou C, Furuya D, Yodh AG et al. Spatiotemporal quantification of cerebral blood flow during functional activation in rat somatosensory cortex using laser-speckle flowmetry. J Cereb Blood Flow Metab 2004; 24: 518–525. 15129183 10.1097/00004647-200405000-00005

[bib34] 34Shin HK, Dunn AK, Jones PB, Boas DA, Moskowitz MA, Ayata C et al. Vasoconstrictive neurovascular coupling during focal ischemic depolarizations. J Cereb Blood Flow Metab 2005; 26: 1018–1030. 16340958 10.1038/sj.jcbfm.9600252

[bib35] 35Paul JS, Luft AR, Yew E, Sheu F-S. Imaging the development of an ischemic core following photochemically induced cortical infarction in rats using Laser Speckle Contrast Analysis (LASCA). NeuroImage 2006; 29: 38–45. 16150612 10.1016/j.neuroimage.2005.07.019

[bib36] 36Li M, Miao P, Yu J, Qiu Y, Zhu Y, Tong S et al. Influences of hypothermia on the cortical blood supply by laser speckle imaging. IEEE Trans Neural Syst Rehabil Eng 2009; 17: 128–134. 19193518 10.1109/TNSRE.2009.2012499

[bib37] 37Devor A, Hillman EM, Tian P, Waeber C, Teng IC, Ruvinskaya L et al. Stimulus-induced changes in blood flow and 2-deoxyglucose uptake dissociate in ipsilateral somatosensory cortex. J Neurosci 2008; 28: 14347–14357. 19118167 10.1523/JNEUROSCI.4307-08.2008 PMC2655308

[bib38] 38Burnett MG, Detre JA, Greenberg JH. Activation–flow coupling during graded cerebral ischemia. Brain Res 2005; 1047: 112–118. 15893740 10.1016/j.brainres.2005.04.024

[bib39] 39Royl G, Leithner C, Sellien H, Müller JP, Megow D, Offenhauser N et al. Functional imaging with Laser Speckle Contrast analysis: Vascular compartment analysis and correlation with Laser Doppler Flowmetry and somatosensory evoked potentials. Brain Res 2006; 1121: 95–103. 17030028 10.1016/j.brainres.2006.08.125

[bib40] 40Takagaki M, Feuerstein D, Kumagai T, Gramer M, Yoshimine T, Graf R et al. Isoflurane suppresses cortical spreading depolarizations compared to propofol—implications for sedation of neurocritical care patients. Exp Neurol 2014; 252: 12–17. 24246282 10.1016/j.expneurol.2013.11.003

[bib41] 41Feuerstein D, Takagaki M, Gramer M, Manning A, Endepols H, Vollmar S et al. Detecting tissue deterioration after brain injury: regional blood flow level versus capacity to raise blood flow. J Cereb Blood Flow Metab 2014; 34: 1117–1127. 24690942 10.1038/jcbfm.2014.53 PMC4083373

[bib42] 42Xie Y, Chen S, Anenberg E, Murphy TH. Resistance of optogenetically evoked motor function to global ischemia and reperfusion in mouse *in vivo*. J Cereb Blood Flow Metab 2013; 33: 1148–1152. 23736644 10.1038/jcbfm.2013.89 PMC3734785

[bib43] 43Kazmi SMS, Salvaggio AJ, Estrada AD, Hemati MA, Shaydyuk NK, Roussakis E et al. Three-dimensional mapping of oxygen tension in cortical arterioles before and afterocclusion. Biomed Opt Express 2013; 4: 1061–1073. 23847732 10.1364/BOE.4.001061 PMC3704088

[bib44] 44Zhang S, Murphy TH. Imaging the impact of cortical microcirculation on synaptic structure and sensory-evoked hemodynamic responses *in vivo*. PLoS Biol 2007; 5: e119. 17456007 10.1371/journal.pbio.0050119 PMC1854912

[bib45] 45Scott NA, Murphy TH. Hemodynamic responses evoked by neuronal stimulation via channelrhodopsin-2 can be independent of intracortical glutamatergic synaptic transmission. PLoS ONE 2012; 7: e29859. 22253807 10.1371/journal.pone.0029859 PMC3254633

[bib46] 46Strong AJ, Anderson PJ, Watts HR, Virley DJ, Lloyd A, Irving EA et al. Peri-infarct depolarizations lead to loss of perfusion in ischaemic gyrencephalic cerebral cortex. Brain 2007; 130: 995–1008. 17438018 10.1093/brain/awl392

[bib47] 47Devor A, Hillman EM, Tian P, Waeber C, Teng IC, Ruvinskaya L et al. Stimulus-induced changes in blood flow and 2-deoxyglucose uptake dissociate in ipsilateral somatosensory cortex. J Neurosci 2008; 28: 14347–14357. 19118167 10.1523/JNEUROSCI.4307-08.2008 PMC2655308

[bib48] 48Bere Z, Obrenovitch TP, Kozák G, Bari F, Farkas E. Imaging reveals the focal area of spreading depolarizations and a variety of hemodynamic responses in a rat microembolic stroke model. J Cereb Blood Flow Metab 2014; 34: 1695–1705. 25074743 10.1038/jcbfm.2014.136 PMC4269732

[bib49] 49Karatas H, Erdener SE, Gursoy-Ozdemir Y, Lule S, Eren-Koçak E, Sen ZD et al. Spreading depression triggers headache by activating neuronal Panx1 channels. Science 2013; 339: 1092–1095. 23449592 10.1126/science.1231897

[bib50] 50Parthasarathy AB, Weber EL, Richards LM, Fox DJ, Dunn AK. Laser speckle contrast imaging of cerebral blood flow in humans during neurosurgery: a pilot clinical study. J Biomed Opt 2010; 15: 066030. 21198204 10.1117/1.3526368 PMC9113397

[bib51] 51Nomura S, Inoue T, Ishihara H, Koizumi H, Suehiro E, Oka F et al. Reliability of laser speckle flow imaging for intraoperative monitoring of cerebral blood flow during cerebrovascular surgery: comparison with cerebral blood flow measurement by single photon emission computed tomography. World Neurosurg 2013; 82: e153–e157. 24047822 10.1016/j.wneu.2013.09.012

[bib52] 52Hecht N, Woitzik J, Dreier JP, Vajkoczy P. Intraoperative monitoring of cerebral blood flow by laser speckle contrast analysis. Neurosurg Focus 2009; 27: E11. 10.3171/2009.8.FOCUS09148 19795950

[bib53] 53Hecht N, Woitzik J, König S, Horn P, Vajkoczy P. Laser speckle imaging allows real-time intraoperative blood flow assessment during neurosurgical procedures. J Cereb Blood Flow Metab 2013; 33: 1000–1007. 23512134 10.1038/jcbfm.2013.42 PMC3705427

[bib54] 54Raabe A, Beck J, Gerlach R, Zimmermann M, Seifert V. Near-infrared indocyanine green video angiography: a new method for intraoperative assessment of vascular flow. Neurosurgery 2003; 52: 132–139. 12493110 10.1097/00006123-200301000-00017

[bib55] 55Woitzik J, Horn P, Vajkoczy P, Schmiedek P. Intraoperative control of extracranial—intracranial bypass patency by near-infrared indocyanine green videoangiography. J Neurosurg 2005; 102: 692–698. 15871512 10.3171/jns.2005.102.4.0692

[bib56] 56De Oliveira JG, Beck J, Seifert V, Teixeira MJ, Raabe A. Assessment of flow in perforating arteries during intracranial aneurysm surgery using intraoperative near-infrared indocyanine green videoangiography. Neurosurgery 2008; 62: 1300–1310. 18695550 10.1227/01.neu.0000333795.21468.d4

[bib57] 57Killory BD, Nakaji P, Gonzales LF, Ponce FA, Wait SD, Spetzler RF et al. Prospective evaluation of surgical microscope-integrated intraoperative near-infrared indocyanine green angiography during cerebral arteriovenous malformation surgery. Neurosurgery 2009; 65: 456–462. 19687689 10.1227/01.NEU.0000346649.48114.3A

[bib58] 58Klijn E, Hulscher HC, Balvers RK, Holland WP, Bakker J, Vincent AJ et al. Laser speckle imaging identification of increases in cortical microcirculatory blood flow induced by motor activity during awake craniotomy. J Neurosurg 2013; 118: 280–286. 23176333 10.3171/2012.10.JNS1219

[bib59] 59Woitzik J, Hecht N, Pinczolits A, Sandow N, Major S, Winkler MK et al. Propagation of cortical spreading depolarization in the human cortex after malignant stroke. Neurology 2013; 80: 1095–1102. 23446683 10.1212/WNL.0b013e3182886932

[bib60] 60Richards LM, Towle EL, Fox J, Douglas J, Dunn AK. Intraoperative laser speckle contrast imaging with retrospective motion correction for quantitative assessment of cerebral blood flow. Neurophotonics 2014; 1: 015006–015006. 26157974 10.1117/1.NPh.1.1.015006 PMC4479045

[bib61] 61Rege A, Murari K, Seifert A, Pathak AP, Thakor NV. Multiexposure laser speckle contrast imaging of the angiogenic microenvironment. J Biomed Opt 2011; 16: 056006. 21639574 10.1117/1.3582334 PMC3124539

[bib62] 62Domoki F, Zölei D, Oláh O, Tóth-Szuki V, Hopp B, Bari F et al. Evaluation of laser-speckle contrast image analysis techniques in the cortical microcirculation of piglets. Microvasc Res 2012; 83: 311–317. 22306444 10.1016/j.mvr.2012.01.003

[bib63] 63Li Y, Baran U, Wang RK. Application of thinned-skull cranial window to mouse cerebral blood flow imaging using optical microangiography. PLoS ONE 2014; 9: e113658. 25426632 10.1371/journal.pone.0113658 PMC4245213

[bib64] 64Atchia Y, Levy H, Dufour S, Levi O. Rapid multiexposure *in vivo* brain imaging system using vertical cavity surface emitting lasers as a light source. Appl Opt 2013; 52: C64–C71. 23458819 10.1364/AO.52.000C64

[bib65] 65Shin HK, Dunn AK, Jones PB, Boas DA, Lo EH, Moskowitz MA et al. Normobaric hyperoxia improves cerebral blood flow and oxygenation, and inhibits peri-infarct depolarizations in experimental focal ischaemia. Brain J Neurol 2007; 130: 1631–1642. 10.1093/brain/awm071 PMC3023418 17468117

[bib66] 66Armitage GA, Todd KG, Shuaib A, Winship IR. Laser speckle contrast imaging of collateral blood flow during acute ischemic stroke. J Cereb Blood Flow Metab 2010; 30: 1432–1436. 20517321 10.1038/jcbfm.2010.73 PMC2949243

[bib67] 67Zhang Q, Zhang L, Yang X, Wan Y, Jia J. The effects of exercise preconditioning on cerebral blood flow change and endothelin-1 expression after cerebral ischemia in rats. J Stroke Cerebrovasc Dis 2014; 23: 1696–1702. 24774439 10.1016/j.jstrokecerebrovasdis.2014.01.016

[bib68] 68Meinke M, Helfmann J, Friebel M, Müller G. Optical properties of platelets and blood plasma and their influence on the optical behavior of whole blood in the visible to near infrared wavelength range. J Biomed Opt 2007; 12: 014024–014029. 17343499 10.1117/1.2435177

[bib69] 69Carp SA, Roche-Labarbe N, Franceschini MA, Srinivasan VJ, Sakadžić S, Boas DA et al. Due to intravascular multiple sequential scattering, Diffuse Correlation Spectroscopy of tissue primarily measures relative red blood cell motion within vessels. Biomed Opt Express 2011; 2: 2047–2054. 21750779 10.1364/BOE.2.002047 PMC3130588

[bib70] 70Kazmi SM, Davis MA, Dunn AK. Go with what flow? interpreting speckle contrast imagery in the multiple scattering limit. Biomedical Optics. Optical Society of America, New York, 2014, BS2B.3.

[bib71] 71Ennos AE Laser speckle and related phenomena 203 (1975). Available at <http://adsabs.harvard.edu/abs/1975lsrp.book.203E>.

[bib72] 72Zhou C, Shimazu T, Durduran T, Luckl J, Kimberg DY, Yu G et al. Acute functional recovery of cerebral blood flow after forebrain ischemia in rat. J Cereb Blood Flow Metab 2008; 28: 1275–1284. 18382471 10.1038/jcbfm.2008.21 PMC2771551

[bib73] 73Kirkpatrick SJ, Duncan DD, Wells-Gray EM. Detrimental effects of speckle-pixel size matching in laser speckle contrast imaging. Opt Lett 2008; 33: 2886–2888. 19079481 10.1364/ol.33.002886

[bib74] 74Miao P, Lu H, Liu Q, Li Y, Tong S. Laser speckle contrast imaging of cerebral blood flow in freely moving animals. J Biomed Opt 2011; 16: 090502. 21950906 10.1117/1.3625231

[bib75] 75Richards LM, Kazmi SMS, Davis JL, Olin KE, Dunn AK. Low-cost laser speckle contrast imaging of blood flow using a webcam. Biomed Opt Express 2013; 4: 2269–2283. 24156082 10.1364/BOE.4.002269 PMC3799684

[bib76] 76Cheng H, Yan Y, Duong TQ. Laser speckle imaging of rat retinal blood flow with hybrid temporal and spatial analysis method 2009; 7163: 716304–716307.

[bib77] 77Lee J, Radhakrishnan H, Wu W, Daneshmand A, Climov M, Ayata C et al. Quantitative imaging of cerebral blood flow velocity and intracellular motility using dynamic light scattering–optical coherence tomography. J Cereb Blood Flow Metab 2013; 33: 819–825. 23403378 10.1038/jcbfm.2013.20 PMC3677104

[bib78] 78Miao P, Rege A, Li N, Thakor NV, Tong S. High resolution cerebral blood flow imaging by registered laser speckle contrast analysis. IEEE Trans Biomed Eng 2010; 57: 1152–1157. 20142159 10.1109/TBME.2009.2037434

